# Postoperative Complications among Major Abdominal Surgeries using Clavien-Dindo Classification in Tertiary Hospital: An Observational Study

**DOI:** 10.31729/jnma.8854

**Published:** 2025-01-31

**Authors:** Manoj Kumar Jha, Kunda Bikram Shah, Anup Thapa, Sunil Basukala, Sumit Kumar Sah

**Affiliations:** 1 Department of Surgery, Shree Birendra Hospital, Chhauni, Kathmandu, Nepal; 2 Nepalese Army Institute of Health Sciences, Sanobharyang, Kathmandu, Nepal

**Keywords:** *Clavien-Dindo classification*, *major abdominal surgeries*, *post-operative complications*

## Abstract

**Introduction:**

The Clavien-Dindo Classification is an easy way to grade any deviation from the postoperative course that is not a normal part of the procedure and does not indicate a failure to achieve the desired cure, regardless of the physician's age or level of competence. We aimed to conduct a this study to determine the prevalence of post-operative complications after major abdominal surgery and to grade them using the Clavien-Dindo Classification.

**Methods:**

This was an observational cross-section study conducted after approval from the Institutional Review Committee (Reference Number: 656). A retrospective data of patients admitted to surgical wards for major surgery from January to June 2022 was included in the study. The postoperative complications were divided as per Clavien-Dindo classification from grade I to grade V. Descriptive statistics was used to analyse data.

**Results:**

Out of 78 patients, 45 (57.69%; 95% CI: 52.69%-62.69%) patients had complication after major abdominal surgery. Among them, 17 (37.78%) patients belonged to age group more than 60 years and 24 (53.33%) patients were female. Amongst the morbidities, diabetes mellitus was seen in 7 (15.56%) patients, hypertension in 6 (13.33%), chronic obstructive pulmonary disease in 5 (11.11%) cases and anaemia in 3 (6.67%) cases. There were 19 (42.22%) patient with Clavien-Dindo Classification Grade II and surgical site infection was observed in 26 (57.78%) cases.

**Conclusions:**

The prevalence of post-operative complication after major abdominal surgery in our study was found to be comparatively higher as compared to similar studies done in similar settings.

## INTRODUCTION

A post-operative complication can be described as any deviation from the expected postoperative recovery that is not a normal part of the procedure and does not indicate a failure to achieve the desired cure, regardless of physician's age or level of competence.^1^ Clavein and Dindo took initiation to grade postoperative complications in 1992 as the "T92 score" and validated on 650 cholecystectomies.^2^ In 2004, the Clavein-Dindo Classification was refined based on previous experience, tested on a large cohort of general surgery patients, and evaluated for reproducibility and acceptability through an international survey.^3^

The classification of post-operative complications is crucial for standardizing surgical outcome data, which enables meaningful comparisons across different centers, therapies, and time periods, ultimately driving improvements in healthcare quality and cost management.^3^

We conducted this study to determine the prevalence of post-operative complications in major abdominal surgeries and to grade them using the Clavien-Dindo Classification for comparison with other studies.

## METHODS

An observational cross-section study was conducted at Department of General surgery, Shree Birendra Hospital, Kathmandu, Nepal. A retrospective data from January to June 2022 was collected after ethical approval from Institutional Review Committee (Reference Number: 656).

A convenience sampling method was used and sample size was calculated using the following formula:


$$\begin{array}{ll} \text{n} & ={\text{Z}}^{2}\times \frac{\text{p}\times \text{q}}{{\text{e}}^{\text{2}}}\\ & =1.96^{2}\times \frac{\text{0.053}\times \text{0.947}}{0.05^{2}}\\ & =78 \end{array}$$

where,

n= minimum required sample size
z= 1.96 at 95% Confidence Interval (CI)
p= prevalence of post-operative complication according to previous comparable study is 5.3%^4^
q= 1 - p

All patients admitted under Department of Surgery, aged more than 18 years, and who underwent major abdominal surgery were included in the study. Major abdominal surgeries include colorectal surgery, hepatobiliary surgery, upper gastrointestinal (GI) surgery (esophageal and stomach surgery), and pancreatic surgery performed under general anesthesia. Exclusion criteria include previously operated abdominal surgery, pregnancy with surgical problems, and complications developed within 30 days after surgery. All patients were evaluated through discharge letter and thorough case sheet revision. Post-operative complications and management were recorded, and postsurgical complication was classified based on Clavien-Dindo classification and assessed.^5^

The Clavien-Dindo classification (CDC) divides the postoperative complication from grade I to grade V, according to the need for treatment. Grade I complications involve deviations from a normal postoperative course that do not require surgical, endoscopic, radiological, or pharmacological treatment, but can include antiemetics, antipyretics, analgesics, diuretics, electrolytes, and physiotherapy, including bedside wound debridement. Grade II complications necessitate pharmacological treatments beyond those allowed in Grade I, and also include blood transfusions or total parenteral nutrition. Grade III complications require surgical, endoscopic, or radiological intervention, with Grade IIIa interventions performed under local anesthesia and Grade IIIb interventions under general anesthesia. Grade IV complications are life-threatening, including central nervous system complication, requiring Intensive Care Unit (ICU) management, with Grade IVa indicating failure of a single organ (including dialysis) and Grade IVb indicating multi-organ failure. Grade V complications include the death of the patient.^3^

All the data were recorded in the proforma of the individual patients. The data were entered, SPSS (Version 16.0) was used for descriptive statistics.

## RESULTS

Out of 78 patients, 45 (57.69%; 95% CI: 52.69%-62.69) patients had complication after major abdominal surgery. There were 24 (53.33%) female and 21 (46.67%) male among patients with complication (Table 1).

**Table 1:** Demographic profile of patients undergoing major abdominal surgery.

	Post-Operative Patients (n=78)	Post-Operative Patients with complication (n=45)
Variable	n(%)	n (%)
**Age**		
<29	11 (14.10)	2 (4.44)
30-39	14 (17.95)	3 (6.67)
40-49	23 (29.49)	13 (28.89)
50-59	11 (14.10)	10 (22.22)
>60	19 (24.36)	17 (37.78)
**Gender**		
Male	44 (56.41)	21 (46.67)
Female	34 (43.59)	24 (53.33)

Amongst the patient having post operative complicaton 19 (42.22%) were in Grade II Clavien- Dindo Classification, 22 (48.89) were ASA Grade 2, Surgical Site Infection (SSI) in 26 (57.78%) patient and Diabetes Mellitus was observed in 7 (15.56%), (Table 2). The median hospital stay was 7 days (IQR:5-11) days.

**Table 2:** Clinical Profile of Post-Operative Patients with Complication (n=45).

Variables	n (%)
**Clavien-Dindo Grades**	
Grade I	13 (28.89)
Grade II	19 (42.22)
Grade IIIa	4 (8.89)
Grade IIIb	3 (6.67)
Grade IVa	2 (4.44)
Grade IVb	2 (4.44)
Grade V	2 (4.44)
**ASA Status**	
Grade 1	12 (26.67)
Grade 2	22 (48.89)
Grade 3	11 (24.44)
Complication^*^	
SSI	26 (57.78)
Fever	11 (24.44)
Nausea/Vomiting	7 (15.56)
Anemia	6 (13.33)
Shock	3 (6.67)
Hemmorhage	2 (4.44)
Anastomotic Leak	2 (4.44)
Poor Nutrition	2 (4.44)
Pneumonia	1 (2.22)
Pain	1 (2.22)
Pelvic Collection	2 (4.44)
Other	6 (13.33)
Comorbidity^*^	
Diabetes mellitus	7 (15.56)
Hypertension	6 (13.33)
Respiratory Disease	5 (11.11)
Anemia	3 (6.67)
Obstructive Jaundice	3 (6.67)
Others	3 (6.67)

ASA=American Society of Anesiotheologist; DM=Diabetis Mellitus; HTN=Hypertension; COPD= Chronic Obstructive Pulmonary Disease; SSI= Surgical Site Infection

* Multiple Choices

There were 6 (13.33%) patients with symptomatic cholelithiasis operated for laparoscopic cholecystectomy, among which all experienced post-operative complication of CDC Grade I (Table 2). Grade IVa complication were seen in 1 (2.22%) case of Carcinoma of Transverse Colon intervened with Extended Hemicolectomy and 1 (2.22%) case of Carcinoma of Rectum intervened with APR. Similarly, Grade IVb complication were seen in 1 (2.22%) case of Carcinoma of stomach operated on with D2 Total Gastrectomy and 1 (2.22%) case of Periampullary Carcinoma operated on with Whipple's Procedure. Grade V complication include 1 (2.22%) case of Whipple's Procedure operated for Periampullary Carcinoma and 1 (2.22%) case of Exploratory Laparotomy with Resection Anastomosis (RA) operated for Mesenteric Ischemia.

**Table 3:** Distribution of Post-Operative Complications by Diagnosis and Surgical Intervention (n=45).

Diagnosis with Surgery	n (%)
Symptomatic Cholelithiasis	6 (13.33)
Laparoscopic Cholecystectomy	6 (13.33)
Carcinoma Rectum	4 (8.89)
Abdominoperineal Resection	2 (4.44)
Anterior Resection	1 (2.22)
Lower Anterior Resection	1 (2.22)
Carcinoma Stomach	4 (8.89)
D2 Distal Gastrectomy	2 (4.44)
D2 Gastrectomy	1 (2.22)
D2 Total Gastrectomy	1 (2.22)
Perforated Duodenal Ulcer	4 (8.89)
Graham Patch Omentopexy	4 (8.89)
Acute Bowel Obstruction	2 (4.44)
Exploratory Laparotomy with Resection Anastomosis	1 (2.22)
Exploratory Laparotomy and release of band	1 (2.22)
Carcinoma Colon	2 (4.44)
Right Hemicolectomy	2 (4.44)
Choledocholithiasis	2 (4.44)
Open CBD Exploration	2 (4.44)
Chronic Calculus Cholecystitis	2 (4.44)
Open Cholecystectomy	2 (4.44)
Chronic Cholecystitis with Contracted Gall Bladder	2 (4.44)
Subtotal Cholecystectomy	2 (4.44)
Hydatid Cyst Liver	2 (4.44)
Laparoscopic Pericystectomy	1 (2.22)
Open Pericystectomy	1 (2.22)
Appendicular Perforation	1 (2.22)
Emergency Appendectomy with Peritoneal Lavage	1 (2.22)
Carcinoma Colon (Transverse)	1 (2.22)
Extended Hemicolectomy	1 (2.22)
Epigastric / Umbilical hernia	1 (2.22)
Onlay Mesh Repair	1 (2.22)
Gall Bladder Perforation	1 (2.22)
Subtotal Cholecystectomy	1 (2.22)
Hypersplenism	1 (2.22)
Splenectomy	1 (2.22)
Mesenteric Cyst	1 (2.22)
Ex. Lap+ Excision of Cyst	1 (2.22)
Mesenteric Ischemia	1 (2.22)
Ex. Lap+ RA	1 (2.22)
Obstructed/Strangulated Hernia	1 (2.22)
Exploration+ hernia repair	1 (2.22)
Periampullary Carcinoma	2 (4.44)
Whipple's Procedure	2 (4.44)
Psoas Abscess	1 (2.22)
Open Incision and Drainage	1 (2.22)
Pyloric Stenosis	1 (2.22)
Gastrojejunostomy	1 (2.22)
Rectal Prolapse	1 (2.22)
Ventral Mesh Rectopexy	1 (2.22)
Sealed DU Perforation	1 (2.22)
Emergency Laparotomy with	1 (2.22)
Peritoneal Lavage	
SMA Syndrome	1 (2.22)
Gastrojejunostomy	1 (2.22)

## DISCUSSION

The prevalence of post-operative complication after major abdominal surgery was found to be 45 (57.69%) which is greater than comparable study done in nepal.^4^ Idriss et al. reported prevalence rate of 21.94%.^5^ Mentula et al. reported prevalence rate of 25.90 % in emergency general surgery while Bolliger et al. reported 12.5% in general surgery.^6,7^ In emergency surgery settings, the prognosis is generally poor and associated with high mortality.^8^ Since we also included emergency cases in our study which may be the cause for higher prevalence of post-operative complication. Additionally, the inclusion of all surgeries rather than limiting to major abdominal surgeries may explain the low prevalence rate of comparable studies.

Among patients with post-operative complication, majority of patients belong to the age group greater than 40 years which is consistent with findings in comparable study reported by Xiao et al. and Bolliger et al.^7,9^ Similarly, Idriss et al. had mentioned mean age group of 39.77 years with range of 1-90 years, with median age of 40 years, having 19.12% patients belonged to over 60-year age and 16.39% patient belonged to 0-16 years.^5^ However, due to exclusion of patients below 18 years, 0-16 years of age had not been included in our study. But higher prevalence of elder age in our study may be due to the more difficult recovery from surgery, which may be caused by decreased physiological reserve. Additionally, advanced age is an independent risk factor for postoperative complications after abdominal surgery, as noted in a cohort study.^10^ Female were more prevalent than male among patients with post-operative complication which is aligned with findings reported by Moeng et al.^11^ But Idriss et al. reported greater prevalence of male among patient with post-operative complication.^5^

The most common complication was SSI accounted for 57.78% among all complication, followed by fever, nausea/vomiting, anemia and shock. Similarly, Idriss et al. also reported SSI as the most prevalent postoperative complication with prevalence of 62.8% among all complication which is also aligned with study conducted by Velickovic et al.^5,12^ Different study found that this increases in SSI after abdominal surgery was observed in people having different risk factors including open surgery, emergency operation, longed operation duration, male sex ,comorbidities including diabetes, and low hemoglobin level.^13,14^ Our study also shows diabetic as most prevalent comorbidities among patients with complication followed by hypertension, COPD and anemia. Similarly, Velickovic et al. mentioned that most post-operative complication had been found on patient who had history of diabetes and lower preoperative hemoglobin level.^12^ The vascular and immunological complication of diabetes might be responsible for post-operative complication including SSI.^15^

Clavien and Dindo initially included length of stay as a criterion for grading post-operative complication.^2^ But due to variations of different governing rules for length of stay in different hospitals, which makes it unreliable to include it in the criteria for grading post-operative complication.^3^ However, the length of stay reflects the severity of post-operative complications; i.e., the more severe the complication, the longer the length of stay.^16^ Velickovic et al. had shown strong correlation between post-operative complication and length of stay.^12^ In our study, the average length of stay among patients with complication was 8.69 days with median of 7 days. Bolliger et al. reported 7.02 days of length of stay with median of 4 days.^7^

Clavein-Dindo Classification (CDC) Grades I and II include only minor deviations from the normal postoperative course that can be treated with drugs, blood transfusions, physiotherapy, and nutrition. In our study, most of post-operative complication belong to grade II, accounted for 42.22%, followed by Grade I, Grade III and Grade IV. This finding is also aligned with comparable studies, reported by Velickovic having Grade II most common with 49.6% among all postoperative complicated cases, and reported by Idriss et al. having Grade II with 82.5 %.^5,12^ However, Moeng et al. reported higher number of Grade I , accounted for 31.6%, followed by Grade IIIb accounted for 26.3%.^11^ Bolliger et al. reported higher number of Grade III complication with 36.2%, followed by Grade I, Grade III and Grade V.^7^

Our study shows CDC Grade I complication among patient with symptomatic cholelithiasis operated for laparoscopic cholecystectomy. Terho et al. mentioned higher prevalence of CDC Grade I and II complication among acute calculous cholecystitis who underwent laparoscopic cholecystectomy.^17^

Our study aligns with Zhang et al., showing a higher incidence of CDC Grade II complications in gastric or colorectal cancer surgeries. Additionally, Zhang et al. found that abdominoperineal resection and distal gastrectomy are associated with more postoperative complications compared to low anterior resection and total gastrectomy, respectively, which aligns with our observations.^18^ However, CDC Grade IVb complications were observed in total gastrectomy in our study, which may be due to higher age, multi-organ resection, and a higher number of lymph nodes retrieved.^19^ Both gastric and colorectal cancer surgery has lower long-term survival outcome if post-operative complication is present.^20,21^

The Clavien-Dindo classification is one of the simplest and easiest way of reporting all complications after surgery regardless of the physicians age or experience. This classification allows surgeons to distinguish between a normal postoperative course from any deviation and the severity of the complication that may be useful for comparing postoperative morbidity in each patient. However, due to the small case number in this study, a definitive statement on the clinical significance of this classification system is not yet possible; however, the promising outcomes should encourage further evaluation in a larger cohort with the goal of potentially establishing its validity as a standard clinical practice.

## CONCLUSIONS

The prevalence of post-operative complication after major abdominal surgery in our study was found to be comparatively higher as compared to studies done amongst all surgical patient and emergency surgeries.

Utilizing Clavien-Dindo classification system, majority of complication were found to be Grade II, with surgical site infection being most common.
